# Genome of the lepidopleurid chiton *Hanleya hanleyi* (Mollusca, Polyplacophora)

**DOI:** 10.12688/f1000research.121706.1

**Published:** 2022-05-23

**Authors:** Rebecca M. Varney, Meghan K. Yap-Chiongco, Nina T. Mikkelsen, Kevin M. Kocot

**Affiliations:** 1 Ecology, Evolution and Marine Biology, University of California, Santa Barbara, Santa Barbara, CA, 93106, USA; 2 Department of Biological Sciences, The University of Alabama, Tuscaloosa, Alabama, 35487, USA; 3 University Museum of Bergen, Univeristy of Bergen, Bergen, 5020, Norway; 4 Alabama Museum of Natural History, The University of Alabama, Tuscaloosa, AL, 35487, USA

**Keywords:** Aculifera, Lepidopleurida, genome, repetitive DNA

## Abstract

Mollusca is the second most species-rich phylum and includes animals as disparate as octopuses, clams, and chitons. Dozens of molluscan genomes are available, but only one representative of the subphylum Aculifera, the sister taxon to all other molluscs, has been sequenced to date, hindering comparative and evolutionary studies. To facilitate evolutionary studies across Mollusca, we sequenced the genome of a second aculiferan mollusc, the lepidopleurid chiton *Hanleya hanleyi* (Bean 1844), using a hybrid approach combining Oxford Nanopore and Illumina reads. After purging redundant haplotigs and removing contamination from this 1.3% heterozygous genome, we produced a 2.5 Gbp haploid assembly (>4X the size of the other chiton genome sequenced to date) with an N50 of 65.0 Kbp. Despite a fragmented assembly, the genome is rather complete (92.0% of BUSCOs detected; 79.4% complete plus 12.6% fragmented). Remarkably, the genome has the highest repeat content of any molluscan genome reported to date (>66%). Our gene annotation pipeline predicted 69,284 gene models (92.9% of BUSCOs detected; 81.8% complete plus 11.1% fragmented) of which 35,362 were supported by transcriptome and/or protein evidence. Phylogenomic analysis recovered Polyplacophora sister to all other sampled molluscs with maximal support. The *Hanleya* genome will be a valuable resource for studies of molluscan biology with diverse potential applications ranging from evolutionary and comparative genomics to molecular ecology.

## Introduction

Mollusca is the second most diverse animal phylum and includes many economically and ecologically important species. Molluscs have been the focus of significant genomic research in recent years, which has enabled exciting comparative and evolutionary genomic investigations (reviewed by Gomes-dos-Santos *et al*. 2020). However, although dozens of molluscan genomes have been sequenced to date, all but one belong to the subphylum Conchifera, which includes the familiar gastropods, bivalves, and cephalopods. The subphylum Aculifera, which includes the eight-shelled chitons (Polyplacophora) and worm-like aplacophorans (Solenogastres and Caudofoveata), is the sister taxon to all other molluscs ( Kocot *et al*. 2020). Surprisingly, just one species from this clade, the chiton *Acanthopleura granulata* (Gmelin, 1791), has been sequenced to date ( Varney *et al*. 2021). Aculiferans are of great interest because as the sister taxon of all other molluscs they are important to understanding molluscan evolution. Further, species in this clade exhibit interesting traits such as iron-hardened teeth ( Brooker and Shaw 2012), a complex armature of scales and spines ( García-Álvarez & Salvini-Plawen 2007), and the only eyes in a living animal with a mineralized lens ( Speiser *et al*. 2011).

Here, we expanded available genomic resources for Aculifera by sequencing and annotating the genome of the chiton *Hanleya hanleyi* (Bean 1844). Extant chitons can be divided into two major clades: Chitonida, the clade represented by the previously published *Acanthopleura* genome, and Lepidopleurida, which includes *Hanleya.* Lepidopleurida is interesting from an evolutionary standpoint as these chitons are thought to be plesiomorphic, with shell features like those of ancient fossil chitons, gills restricted to the posterior region of the body, and simple gamete structure ( Sigwart *et al*. 2011). Because of this suite of putatively ancestral characteristics and its phylogenetic position as the sister taxon to all other chitons, Lepidopleurida is thought to be critical to understanding large-scale patterns in molluscan evolution ( Sigwart 2008). *Hanleya hanleyi* is a widely distributed, sponge-feeding lepidopleurid that is relatively common off Bergen, Norway and it is the largest lepididopleurid chiton known ( Sirenko *et al*. 2016), making it an excellent choice for genome sequencing.

## Methods

**Figure 1.**
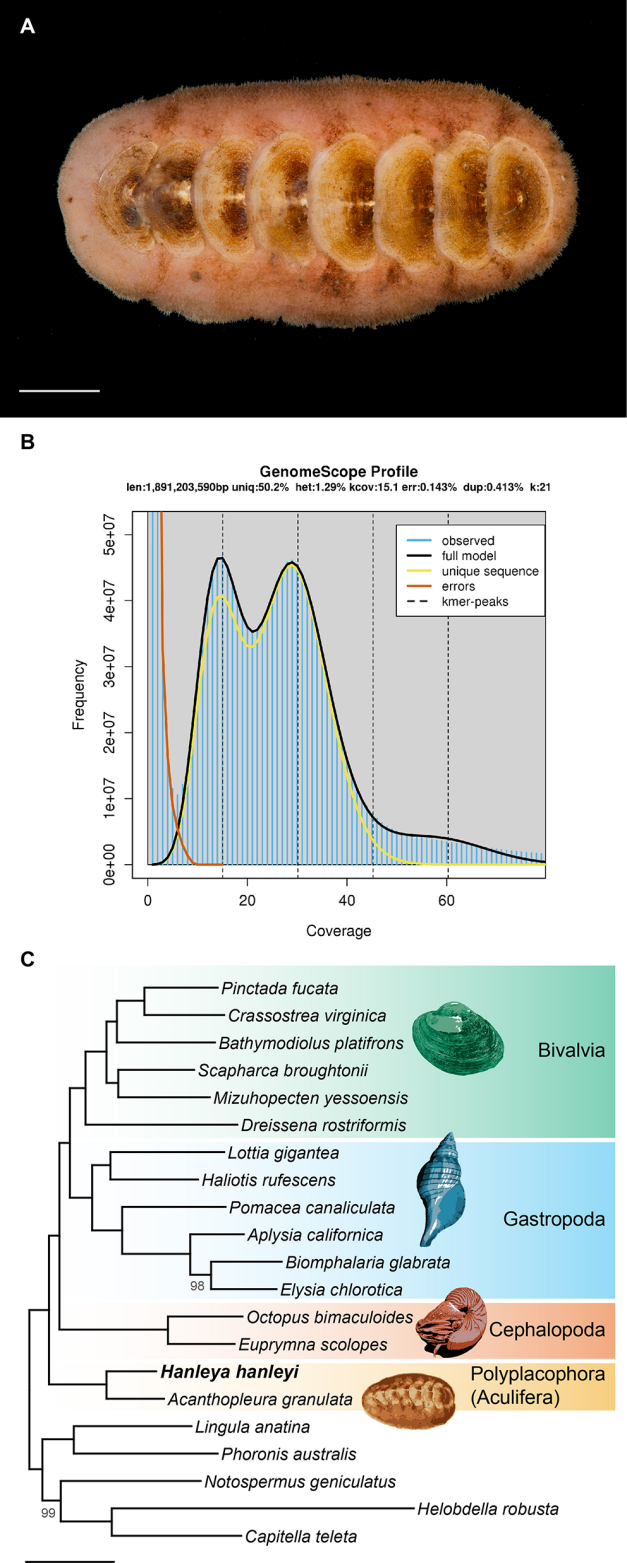
A. Specimen of *Hanleya hanleyi* used for genome sequencing (ZMBN 146951). Scale bar = 8 mm. B. GenomeScope analysis of the paired-end Illumina data. The presence of two peaks indicates that Hanleya has a diploid genome, as expected. Heterozygosity is measured via k-mer distribution (presented at top of graph as “het”). C. Phylogenetic analysis of 2,331 nuclear protein-coding genes. Bootstrap support values below 100 are displayed at each node. Scale bar = 0.2 substitutions per site.

The specimen of *Hanleya hanleyi* used for genome sequencing ( Figure 1A) was collected by N.T.M. off Bergen, Norway in 2018 and is deposited in the University Museum of Bergen under catalog number ZMBN 146951. The genome was sequenced with a combination of short and long reads. To produce short-read data, genomic DNA was extracted from 96% ethanol-preserved samples of foot tissue using a CTAB-phenol-chloroform method following Varney *et al*. (2021). A sequencing library was prepared in-house using the Illumina TruSeq DNA PCR-Free kit with dual indexing according to the manufacturer’s instructions. This library was sequenced by Macrogen USA on one lane of the Illumina HiSeq X instrument with 150 bp paired-end (PE) sequencing. To produce long-read data via Oxford Nanopore sequencing, genomic DNA was extracted with an EZNA Tissue DNA Kit (Omega Bio-tek) and cleaned and enriched for high-molecular-weight fragments with the Short-Read Eliminator kit (Circulomics) according to the manufacturer’s instructions. Three sequencing libraries were prepared with the LSK-109 ligation-based library preparation kit and sequenced in-house on three R9.4.1RevD flow cells on a GridION. Reads were base called with Guppy 4.0 and trimmed with PoreChop ( Wick 2018) with the --discard_middle flag.

A different specimen of *Hanleya hanleyi* collected by dredging near Bergen, Norway in summer 2008 was gifted to the authors by Dr. Hans Torre Rapp for transcriptome sequencing and is deposited in the Alabama Museum of Natural History under catalog number ALMNH:Inv:23399. Notably, tissue from this same individual was used to generate the 454 pyrosequencing-based foot tissue transcriptome for this species (SRR108987) published by Kocot *et al*. (2011). For Illumina transcriptome sequencing, RNA extraction was performed on mantle tissue preserved in RNAlater and stored at -80°C using the Omega Bio-tek EZNA Mollusc RNA Extraction Kit with an on-column DNAse digestion. RNA concentration was measured using a Qubit 3.0 (Thermo Fisher) fluorometer with the RNA High Sensitivity kit, RNA purity was assessed by measuring the 260/280 nm absorbance ratio using a Nanodrop Lite (Thermo Fisher), and RNA integrity was evaluated using a 1% SB agarose gel. RNA was sent to Psomagen (Cambridge, MA, USA) for Illumina TruSeq RNA v2 library preparation (polyA enrichment) and sequencing on the Illumina HiSeq 2500 system with 100 bp PE sequencing.

Genome size and heterozygosity were estimated based on the PE Illumina reads using GenomeScope 2 ( Ranallo-Benavidez *et al*. 2020) with a k-mer of 21. Hybrid genome assembly was performed with MaSuRCA 3.3.5 ( Zimin *et al*. 2017), which consolidates PE data into super reads and then uses long-read data to scaffold and gap-fill. Recommended settings for eukaryotes with >20X Illumina coverage and “PE= pe 587 88” were used. At this point (and after each step involving filtering or polishing the genome assembly; see below), we assessed assembly quality with QUAST 5.0.2 ( Mikheenko *et al*. 2018) and completeness with BUSCO 5.1.3 ( Manni *et al*. 2021) using the Metazoa odb_10 dataset and the “--long” flag. We then removed redundant haplotigs with purge_dups. Finally, the remaining scaffolds were polished with POLCA ( Zimin & Salzberg 2020) using the Illumina paired-end reads, which were first quality- and adapter-trimmed with trimmomatic 1.8.0 ( Bolger *et al*. 2014) using the following settings: “ILUMINACLIP:adapters.fasta:2:30:10 LEADING 10 TRAILING 10 SLIDINGWINDOW:4:15 MINLEN:50.”

Contamination was then screened for and removed with BlobTools2 ( Challis *et al*. 2020). The POLCA-polished assembly was searched against the Uniprot reference proteomes (02-Jun-2021 release) with Diamond 2.0.14 ( Buchfink *et al*. 2015) using the following settings: “--sensitive --index-chunks 1 --block-size 10 --max-target-seqs 1 -evalue 1e-25 --outfmt 6.” The quality- and adapter-trimmed genomic PE reads were then mapped to the genome with minimap 2.23 ( Li 2018) with the following settings: “-ax sr.” The output of these tools as well as full_table.tsv generated by BUSCO were then used as input files to run BlobTools2. We removed scaffolds with fewer than 10 mapping Illumina reads, scaffolds not annotated as Metazoa, and scaffolds with a GC content <0.30 or >0.55, which appeared as clear outliers when GC content was plotted against coverage.

For genome annotation, repeats in the final contamination-filtered assembly were annotated and softmasked with RepeatMasker using a custom repeat database generated with RepeatModeler ( Smit & Hubley 2015). For RepeatModeler, a maximum genome sample size of 1M and the --LTRStruct option were used. For RepeatMasker, the slow and gccalc options were used. The engine used for both programs was rmblast. Available chiton and select other mollusc proteomes (see data on Dryad for details) were then mapped to the final genome assembly with ProtHint 2.6 ( Brůna *et al*. 2020) with an e-value cutoff of 1e-25. We ran TrimGalore (Krueger *et al*. 2021) on the transcriptome reads with the following settings: “-q 30 --illumina --trim-n.” The trimmed and filtered transcriptome reads were then mapped to the genome using STAR 2.4.0k ( Dobin *et al*. 2013) with “--genomeChrBinNbits 15 --chimSegmentMin 50.” Annotation of protein-coding genes was performed with BRAKER 2.1.6 (Bruna *et al*. 2021) using the output of ProtHint and STAR with the following settings: “--eptmode --softmasking --crf.” Predicted transcripts with at least partial support from the *Hanleya* transcriptome and/or other chiton proteomes were identified with the selectSupportedSubsets.py bundled with BRAKER.

Building on the phylogenomic analysis of Varney *et al*. (2021), we identified homologous protein sequences in the full set of *Hanleya hanleyi* gene models (including those with no transcript or protein evidence) to the complete proteome of the only other available chiton genome, *Acanthopleura granulata*, and the proteomes of 19 other lophotrochozoans, including 14 other molluscs, 2 annelids, 1 brachiopod, 1 phoronid, and 1 nemertean using OrthoFinder 2.4.0 ( Emms & Kelly 2019). We then identified orthologous genes from the homogroups produced by OrthoFinder using the pipeline of Varney *et al*. (2021) except we retained only genes sampled for 18/21 taxa using PhyloPyPruner. Phylogenetic analysis on the concatenated supermatrix in IQ-Tree 2.1.3 ( Minh *et al*. 2020) using the best-fitting model for each partition (-m MFP). The tree was arbitrarily rooted with all non-molluscan taxa.

## Results

Illumina transcriptome sequencing yielded 25.8M reads or 5.8 Gbp. For the genome, Oxford Nanopore sequencing of three flowcells yielded 13.30, 12.47, and 13.91 Gbp (4,401,106, 4,551,630, and 7,027,597 reads respectively) and Illumina sequencing yielded 129 Gbp (860,037,886 reads). GenomeScope analysis of the PE genomic data inferred a genome size of 1.89 Gbp and a heterozygosity of 1.3% ( Figure 1B). Assembly with MaSuRCA yielded an initial assembly consisting of 81,742 scaffolds totaling 3.11 Gbp with an N50 of 59.9 Kbp. After polishing and purging redundant haplotigs, the assembly was reduced to 62,284 scaffolds totaling 2.77 Gbp with an N50 of 66.1 Kbp. Despite being somewhat fragmented, the resulting assembly is rather complete with 94.9% of BUSCOs detected (83.3% complete plus 11.6% fragmented). After removing putative contaminant scaffolds – those with fewer than 10 mapping Illumina reads, not annotated as Metazoa (Proteobacteria, Firmicutes, and “Bacteria-undef”) or as “no-hit” in BlobTools, and/or with a GC content <0.30 or >0.55 – the final assembly consisted of 57,495 scaffolds totaling 2.52 Gbp with an N50 of 65.0 Kbp, an N90 of 19.97 Kbp, an L50 of 10.42 Kbp, an L90 of 38.44 Kbp, and a longest scaffold of 0.8 Mbp. After removal of putative contamination, 92.0% of BUSCOs could be detected (79.4% complete [74.4% single-copy and 5.0% duplicated], 12.6% fragmented, and 8.0% missing).

At 2.5 Gbp, the *Hanleya hanleyi* genome is over four times the size of that of the only other chiton with a genome sequenced to date, *Acanthopleura granulata.* RepeatModeler identified 327 families of repeats across five major classes ( Table 1). The diversity of repetitive DNA motifs in the *Hanleya* genome is on par with that of other molluscan genomes with the exception of long terminal repeats (LTRs), which are much more diverse (100 different types) in *Hanleya* than any other molluscan genome we examined. A majority of repeats were annotated by RepeatClassifier as unclassified, likely because there are still few molluscan genomes incorporated in repetitive element databases. The genome of *Hanleya* has more than double the total repetitive content of that of *Acanthopleura*: 66.41% total interspersed repeats in *Hanleya* compared to 23.56% in *Acanthopleura* (Varney *et al*. 2020). Moreover, to our knowledge, the genome of *Hanleya* has an overall repetitive content higher than *any* mollusc sequenced to date ( Gomes-dos-Santos *et al*. 2020).

**Table 1.** The number of repetitive elements of various types across several molluscan genomes as indicated by RepeatModeler.

Repetitive element	*Hanleya hanleyi*	*Acanthopleura granulata*	*Haliotis rufescens*	*Pinctada fucata*	*Crassostrea virginica*	*Bathymodiolus platifrons*	*Scapharca broughtonii*	*Lottia gigantea*
buffer	0	4	1	3	0	0	1	2
DNA	65	76	154	132	322	224	186	108
LINE	151	44	119	161	78	156	81	49
SINE	7	23	13	16	13	7	32	26
LTR	100	22	31	21	47	38	19	21
RC	4	10	13	0	85	52	60	3
Satellite	0	4	10	0	2	7	7	1

BRAKER predicted 69,284 gene models with 92.9% of BUSCOs detected (81.8% complete [75.6% single-copy and 6.2% duplicated], 11.1% fragmented, and 7.1% missing). Of these, 35,362 were supported by transcriptome and/or protein evidence. Removal of gene models not supported by transcriptome or protein evidence had little effect on the estimated completeness of the gene models as 92.2% of BUSCOs were detected (81.3% complete [75.2% single-copy and 6.1% duplicated] 10.9% fragmented, and 7.8% missing).

Comparison of the full set of *Hanleya* gene models to the gene models from 20 other lophotrochozoans in OrthoFinder resulted in 185,272 groups of homologous sequences. Our pipeline selected 2,331 single-copy genes sampled for at least 18 of the 21 taxa. Of these, *Hanleya* was sampled for 2,168 genes (93%), further demonstrating the completeness of this genome. For comparison, *Lottia gigantea* (Gastropoda) was sampled for 2,243, *Crassostrea virginica* (Bivalvia) was sampled for 2,076, and *Acanthopleura granulata* (Polyplacophora) was sampled for 1,999. Concatenation resulted in a supermatrix 831,793 amino acids in length with 16.7% missing data. Phylogenetic analysis resulted in a strongly supported tree with maximal support for Polyplacophora and placement of Polyplacophora as sister to all other sampled molluscs ( Figure 1C).

Sequencing data has been uploaded to NCBI SRA (see *Underlying data)* and all other results to Figshare (see *Extended data* ( Kocot 2022)).

## Conclusions

Despite challenges in assembling this relatively large (2.5 Gbp), heterozygous (1.3%), and repetitive (66.4%) genome, BUSCO analysis indicates that it is rather complete with 92.0% of BUSCOs detected in the final, decontaminated genome and 92.9% and 92.2% of BUSCOs detected in the full and evidence-supported predicted transcript sets, respectively. Our orthology inference pipeline recovered 93% of the genes sampled from at least 18/21 lophotrochozoan genomes in the *Hanleya*, further supporting the near completeness of this genome.

## Data availability

### Underlying data

NCBI Sequence Read Archive (SRA): RNA-Seq of *Hanleya hanleyi* mantle. Accession number SRX8235059. https://identifiers.org/ncbiprotein:SRX8235059.

NCBI SRA: Illumina Sequencing of *Hanleya hanleyi* gDNA. Accession number SRR18273088. https://identifiers.org/ncbiprotein:SRR18273088.

NCBI SRA: GridION Sequencing of *Hanleya hanleyi* gDNA. Accession numbers SRX14411365, https://identifiers.org/ncbiprotein:SRX14411365; SRX14411366, https://identifiers.org/ncbiprotein:SRX14411366; and SRX14411367, https://identifiers.org/ncbiprotein:SRX14411367.

### Extended data

Figshare: *Hanleya hanleyi* genome extended data. https://doi.org/10.6084/m9.figshare.19672449.v2 ( Kocot 2022).

This project contains the following extended data:

- 01_Jellyfish_and_GenomeScope.zip (Jellyfish and GenomeScope results)
- 02_MaSuRCA.zip (genome assembly produced by MaSuRCA)
- 03_purge_dups.zip (heterozygosity-purged genome assembly)
- 04_POLCA.zip (purge_dups output polished with Illumina reads in POLCA)
- 05_QUAST_and_BUSCO_on_final_genome_assembly.zip (QC of final assembly after POLCA)
- 06_RepeatMasker_and_RepeatModeler.zip (RepeatMasker & RepeatModeler output)
- 07_BlobTools.zip (BlobTools contamination screening results)
- 08_BRAKER.zip (Genome annotation with BRAKER)
- 09_BUSCO_on_gene_models.zip (QC on final gene models produced by BRAKER)
- final_genome_assembly_and_annotations.zip (final genome assembly and annotation)

Data are available under the terms of the Creative Commons Attribution 4.0 International license (CC-BY 4.0).

## Ethical approval

Ethics permits were not required to undertake this research because Institutional Animal Care and Use Committee (IACUC) review is not required for use of invertebrates in research activities at the University of Alabama.
