# Supplementary material for: Genome of the lepidopleurid chiton Hanleya hanleyi (Mollusca, Polyplacophora)
Source: F1000Res. 2022 May 23;11:555. [Version 1] doi: 10.12688/f1000research.121706.1 (PMC9366239; doi:10.12688/f1000research.121706.1)
Supplement: Peer review history

# Peer review: Reviewer response for version 1

**DOI:** 10.5256/f1000research.133601.r145650

**Published:** 2022-08-10

**Authors:** Samuel Abalde

**Affiliations:** 1 Department of Zoology, Swedish Museum of Natural History, Stockholm, Sweden

Mollusca is an important animal phylum, and as such it has received a lot of attention from the scientific community. However, it is important to note that much of this attention has been drawn towards the three most diverse and economically important molluscan classes, while all other have been relatively neglected. From a genomic perspective the scenario is similar, with all but two mollusk genomes published to date sequenced from the same three classes. Despite the importance of the Aculifera, the clade containing chitons and aplacophorans, one of the two main clades of mollusks and hence fundamental to fully understanding mollusk evolution, only one genome has been generated to date, hampering comparative studies.

 In this manuscript, Varney et al. report the complete genome of the chiton *Hanleya hanleyi*, the second aculiferan (and chiton) genome. The genome is relatively fragmented but seems to be very complete, and it will become an important addition to future studies of mollusk evolution.

 I would like to congratulate the authors for their work. The manuscript is concise but it presents all the relevant information and the methods look sound. I have only three minor comments that, although will not change substantially the manuscript, I think the authors should consider:

- **“Extant chitons can be divided into two major clades: Chitonida, the clade represented by the previously published *Acanthopleura* genome, and Lepidopleurida, which includes *Hanleya.*”** I am not an expert on chiton systematics, but to the best of my knowledge there are three main groups: Callochitonida, Chitonida, and Lepidopleurida. The same three groups were recovered in a recent phylogeny ^1^. I am not aware of more recent updates on this matter, but if so then I think this should be referenced in the text to avoid misunderstandings.
- Pertaining to the previous comment: **“Because of this suite of putatively ancestral characteristics and its phylogenetic position as the sister taxon to all other chitons, Lepidopleurida is thought to be critical to understanding large-scale patterns in molluscan evolution.” **If we accept there are only two main groups, then Lepidopleurida is as sister to all other chitons as Chitonida, so this sentence is technically correct but misleading, because it makes you think of a ladderized tree and not in a sister relationship. If we consider the three groups mentioned above, then this sentence is correct.
- As for the repeat content, I wonder about their distribution in the genome. This number is not high enough to rise suspicions, there are other genomes above 50%, but since it will set the new upper limit for repeat content in molluscan genomes I would like to double check this figure is correct. Are the repeats scattered around the genome? Is it possible that they might be concentrated in a few contigs that should be quality-checked?

Are the datasets clearly presented in a usable and accessible format, and the assembly and annotation available in an appropriate subject-specific repository?

Yes

Are sufficient details of the sequencing and extraction, software used, and materials provided to allow replication by others?

Yes

Are the rationale for sequencing the genome and the species significance clearly described?

Yes

Are the protocols appropriate and is the work technically sound?

Yes

Reviewer Expertise:

Phylogenomics, Bioinformatics, Genomics, Invertebrate Biology

I confirm that I have read this submission and believe that I have an appropriate level of expertise to confirm that it is of an acceptable scientific standard.

## References

[1] : A mitogenomic phylogeny of chitons (Mollusca: Polyplacophora). *BMC Evolutionary Biology* .2020;20(1) : 10.1186/s12862-019-1573-2 10.1186/s12862-019-1573-2 32024460PMC7003433

# Peer review: Reviewer response for version 1

**DOI:** 10.5256/f1000research.133601.r138695

**Published:** 2022-06-24

**Authors:** Vanessa L. Gonzalez

**Affiliations:** 1 Global Genome Initiative, National Museum of Natural History, Smithsonian Institution, Washington, DC, USA

The manuscript is clear, concise, well written and scientifically sound. This phylogenetically important taxon’s genome assembly is a much-needed addition to the currently sampling of available molluscan genomes. All methods are explicitly outlined and are appropriate for the genome assembly (hybrid assembly, annotation & phylogenetic methods). The outcome of the annotation process is expected with the resulting contiguity of the genome (fragmented).

 Not sure if I have missed it in the text, but is seems as though the database and database version that was used to calculate the BUSCO scores is not listed (maybe Metazoa?). If it was the Metazoan database, I think it would be helpful to also add the BUSCO scores for the Molluscan specific database as well.

Are the datasets clearly presented in a usable and accessible format, and the assembly and annotation available in an appropriate subject-specific repository?

Yes

Are sufficient details of the sequencing and extraction, software used, and materials provided to allow replication by others?

Yes

Are the rationale for sequencing the genome and the species significance clearly described?

Yes

Are the protocols appropriate and is the work technically sound?

Yes

Reviewer Expertise:

Biodiversity Genomics, Invertebrate Biology, Bioinformatics

I confirm that I have read this submission and believe that I have an appropriate level of expertise to confirm that it is of an acceptable scientific standard.
